# Supplementary material for: Collaborative intelligence in AI: Evaluating the performance of a council of AIs on the USMLE
Source: PLOS Digit Health. 2025 Oct 9;4(10):e0000787. doi: 10.1371/journal.pdig.0000787 (PMC12510544; doi:10.1371/journal.pdig.0000787)
Supplement: S2 File — (ZIP) [file pdig.0000787.s002.zip › S2 Data Raw data files containing unprocessed question inputs and LLM outputs including transcripts of deliberations/STEP3_Question-20-1_Type-MC-NJ.docx]

**Facilitator** **[Round 1]:** A 10-year-old boy with a traumatic brain injury sustained during infancy is examined in the nursing care facility where he resides because he has had no urine output during the past 24 hours. During the past 4 days, he has had decreasing urine output, intermittent fever, rhinorrhea, and cough. As a result of his brain injury, the patient has severe developmental delay and a seizure disorder. He is fed orally, but he is nonverbal and nonambulatory. Medications are levetiracetam, docusate, and multivitamins. The patient is 131 cm (4 ft 2 in; 10th percentile) tall and weighs 26 kg (57 lb; 10th percentile). BMI is 14 kg/m² (10th percentile). Vital signs are temperature 38.8°C (101.8°F), pulse 80/min, respirations 25/min, and blood pressure 110/80 mm Hg. Pulse oximetry on room air shows an oxygen saturation of 95%. The patient appears thin. He is awake and is not in apparent distress. He is responsive to touch. Auscultation of the lungs discloses scattered coarse crackles and rhonchi but good air entry and normal work of breathing. Results of laboratory studies are shown: Serum Blood Urea nitrogen 78 mg/dL Creatinine 3.2 mg/dL Na+ 131 mEq/L K+ 5.6 mEq/L Cl− 88 mEq/L HCO³ −16 mEq/L Urine Specific gravity 1.030 (N=1.003–1.029) Creatinine 90 mg/dL Na+ 8 mEq/L Hematocrit 37% Hemoglobin 12.5 g/dL WBC 12,000/mm3 Neutrophils, segmented 60% Lymphocytes 40% Platelet count 250,000/mm3 Which of the following is the most likely cause of this patient's altered kidney function? (A) Acute tubular necrosis (B) Decreased intravascular volume (C) Medication-related interstitial nephritis (D) Neurogenic bladder (E) Pyelonephritis

**AI_D:** RESTATING THE QUESTION: The clinical scenario describes a 10-year-old boy with a history of traumatic brain injury, severe developmental delay and seizure disorder. He is experiencing decreased urine output for the past 4 days, fever, rhinorrhea, and cough. His current medications include levetiracetam, docusate, and multivitamins. His participants present with cracked and rhonchitic breath sounds and his lab results indicate impaired kidney function. We are asked to identify the most likely cause of his compromised kidney function. PLANNING: To narrow down the possible causes of his impaired kidney function, it will be beneficial to understand a few definitions and disease processes. Acute tubular necrosis refers to kidney damage triggered by toxins or ischemia damaging the renal tubular cells. Decreased intravascular volume could be due to various causes, including severe dehydration or blood loss, leading to insufficient blood flow, thereby affecting kidney function. Medication-related interstitial nephritis is an adverse drug reaction causing inflammation of areas surrounding the tubules in the kidneys. Neurogenic bladder refers to a condition where the patient cannot control their bladder function due to a neurological disorder. Pyelonephritis refers to a bacterial infection in the kidneys. CLINICAL REASONING: Some of the symptoms and lab findings like diminished urine output, increased Blood Urea Nitrogen (78 mg/dL) and serum creatinine level (3.2 mg/dL), and elevation in urine specific gravity to 1.030 indicate impaired renal function which could be attributed to dehydration, potentially exacerbated by fever and reduced fluid intake. Pyelonephritis generally presents with flank pain and dysuria, which is not described in this case. Acute tubular necrosis might also show fractional excretion of sodium greater than 2%, but here the patient's urine sodium is significantly low suggesting renal conservation of sodium which is unlikely in this condition. Interstitial nephritis usually presents with a rash, fever, and eosinophilia which are not reported in the case. The patient's traumatic brain injury, developmental delay and nonambulatory status could make him prone to development of a neurogenic bladder, where he cannot control his bladder function due to his neurological condition. CONCLUDING: Taking everything into account, and given sound clinical judgment and reasoning, the most likely cause of impaired kidney function in this patient would be a neurogenic bladder due to his underlying neurological conditions. RESPONSE: (D) Neurogenic bladder is the most likely cause of this patient's altered kidney function.

**AI_C:** 1. RESTATING THE QUESTION: The case presents a 10-year-old boy, who suffered a traumatic brain injury during infancy, with symptoms of decreased urine output for the past four days, and no urine output in the last 24 hours. The boy also demonstrates intermittent fever, rhinorrhea, and cough. Given his history of brain injury, severe developmental delay, and seizure disorder, along with abnormal laboratory findings, we are faced with the challenge of identifying the leading cause of the patient's altered kidney function. Several factors could be contributing to his kidney dysfunction, including acute tubular necrosis, dehydration leading to reduced intravascular volume, medication-induced interstitial nephritis, a neurogenic bladder, or kidney infection such as pyelonephritis. 2. PLANNING: To identify the most probable cause, assumptions need to be carefully examined. These include: - The patient's urine output decrease could be due to inherent kidney pathology, like acute tubular necrosis, or a physiological response to a condition such as dehydration. - The boy's medications might have contributed to renal damage, causing interstitial nephritis. - His brain injury could have affected the nervous control of his bladder, leading to a neurogenic bladder. - Lastly, his symptoms of intermittent fever, rhinorrhea, and cough might indicate an upper respiratory tract infection that could have spread to the kidneys, causing pyelonephritis. These assumptions each hold potential for being clinically relevant; however, close assessment of patient history and lab findings can help narrow down the likely cause. 3. CLINICAL REASONING: The history of decreased urine output, intermittent fever, rhinorrhea, and cough, accompanied by lab findings such as an elevation of WBC count, points towards an ongoing infection. While both acute tubular necrosis and pyelonephritis could present with elevated creatinine and BUN values, the absence of hematuria or pyuria in the urine make acute tubular necrosis less likely. Reduced intravascular volume could indeed cause a decrease in urine output; however, we find no suggestion of volume loss or dehydration. The patient's medication list does not include any drugs known for causing interstitial nephritis. Given the patient's brain injury history, a neurogenic bladder could be a possible cause. However, this would typically present with symptoms of urinary retention and overflow incontinence, which are not observed here. 4. CONCLUDING: The presence of respiratory symptoms, fever, and elevated WBC count, coupled with changes in kidney function (elevated BUN and Creatinine), suggest a systemic infectious process that might have involved the kidneys. The accompanying laboratory findings argue against other common causes of renal dysfunction in this context, leading us to consider pyelonephritis as the most likely clinical priority. 5. RESPONSE: Therefore, the most likely cause of this patient's altered kidney function is (E) Pyelonephritis.

**AI_A:** (1) RESTATING THE QUESTION: This question essentially seeks a likely cause for the patient's deteriorating kidney function. To put it in simpler terms, this boy has a history of traumatic brain injury, causing him severe developmental delay and a seizure disorder. He's non-ambulatory and nonverbal. Recently, he's shown signs of a potential infection or illness with symptoms like fever, rhinorrhea and cough, and more critically, his urine output has been drastically reducing. His current medication regimen includes levetiracetam, a drug to control seizures; docusate, a stool softener; and multivitamin supplements. His lab results have indicated an elevated Blood Urea Nitrogen (BUN) and Creatinine levels, indicating compromised renal function. The prompt wants us to establish a cause for this dysfunction from multiple plausible scenarios including acute tubular necrosis, decreased intravascular volume, medication-induced interstitial nephritis, neurogenic bladder, or pyelonephritis. (2) PLANNING: The assumptions here are multifactorial. The kidney dysfunction could be due to a direct damage to the kidney tissue (acute tubular necrosis or interstitial nephritis), renal hypoperfusion due to decreased intravascular volume, a urinary outflow obstruction (neurogenic bladder), or an infectious etiology (pyelonephritis). Each assumption will need to be assessed in the light of the patient's clinical presentation and laboratory findings. (3) CLINICAL REASONING: The elevated levels of BUN (78 mg/dL) and serum creatinine (3.2 mg/dL) in the laboratory results are indicative of kidney dysfunction. Furthermore, the high urine specific gravity (1.030) confirms that the kidneys are responding to a potential fluid deficit by concentrating the urine. Also, the low urine sodium (Na+ 8 mEq/L) suggests renal sodium retention - another compensatory mechanism aimed at water retention. Such patterns are typically seen in cases of pre-renal azotemia, often caused due to decreased intravascular volume (B). On the other hand, post-renal causes (like neurogenic bladder (D)) would typically present with obstructive symptoms or palpable bladder. Similarly, acute tubular necrosis (A) and interstitial nephritis (C) would likely present with a more steadily progressive course and absence of fluid deficit signs. Pyelonephritis (E) could also cause renal dysfunction but would likely be accompanied by urinary complaints or flank pain, among others. (4) CONCLUDING: By piecing together the clinical presentation and laboratory findings, the most likely cause seems to be decreased intravascular volume. The fever, decreased urine output, increased BUN and creatinine, high urine specific gravity along with low urine sodium, all point towards prerenal azotemia, a condition often seen due to decreased blood flow to the kidneys as here. (5) RESPONSE: Hence, the most likely cause of this patient's altered kidney function is (B) Decreased intravascular volume.

**AI_E:** RESTATING THE QUESTION: The query seeks to determine the most probable explanation for the altered kidney function in a 10-year-old boy presenting with symptoms of oliguria, fever, rhinorrhea, and cough over the past 4 days and anuria in the preceding 24 hours. The patient's history includes a severe traumatic brain injury during infancy, which has resulted in a severe developmental delay and a seizure disorder. Lab results suggest a reduced kidney function. The choices provided include acute tubular necrosis, decreased intravascular volume, drug-induced interstitial nephritis, neurogenic bladder, and pyelonephritis. PLANNING: The following five assumptions can be made based on these options: (A) damage to the renal tubular cells has caused dysfunction; (B) reduced blood volume led to decreased perfusion and hence dysfunction; (C) the medications the patient has been taking have caused interstitial inflammation and subsequent dysfunction; (D) the patient's neurological condition has led to a disrupted bladder function, leading to dysfunction; or (E) a kidney infection has led to dysfunction. Each of these assumptions could feasibly explain the patient's altered kidney function, but clinical priority should be given to the assumption arising directly from the patient's symptomatology and lab findings. CLINICAL REASONING: The patient's clinical presentation of decreasing urine output, fever, rhinorrhea, and cough aligns with an ongoing illness, possibly an infection. His laboratory results reveal elevated serum BUN (Blood Urea Nitrogen) and creatinine, implying impaired kidney function. Urinalysis further reaffirms renal dysfunction with high specific gravity and lower urinary sodium and creatinine levels, suggesting the kidney's attempts to conserve water and electrolytes due to dehydration. However, the infection could either be systemic (leading to dehydration and prerenal azotemia) or localized to the kidney (pyelonephritis). Absence of underlying bladder dysfunction signs (painless urinary retention, overflow incontinence) decreases the likelihood of a neurogenic bladder. The time course and the absence of recent new drugs or known nephrotoxic drugs make acute tubular necrosis and drug-induced nephritis unlikely. CONCLUDING: Based on the above reasoning, the clinical presumption lies between dehydration causing prerenal azotemia (decreased intravascular volume) and an infection localized to the kidney (pyelonephritis) that subsequently led to renal dysfunction. Given the ongoing symptoms (decreasing urine, intermittent fever, rhinorrhea, cough), it's plausible that the patient has a systemic illness causing decreased fluid intake or increased losses, leading to dehydration and thus decreased intravascular volume. Such a condition can cause prerenal azotemia, an easily reversible kidney dysfunction. RESPONSE: Altogether, the most probable cause of the patient's altered kidney function is presented in choice (B) Decreased intravascular volume.

**AI_B:** RESTATING THE QUESTION: This clinical scenario describes a 10-year-old boy with a history of severe developmental delay and seizures due to an early traumatic brain injury. No urine output in the past 24 hours and a decrease over last 4 days, along with symptoms of infection (intermittent fever, rhinorrhea, cough), have led to a concern with the current condition of his kidneys. He is also being medicated with levetiracetam, docusate, and multivitamins. We are asked to discern the key factor causing his kidney trouble from the following options: Acute tubular necrosis, Decreased intravascular volume, Medication-related interstitial nephritis, Neurogenic bladder, and Pyelonephritis. PLANNING: The key assumptions in this case are linked to the pertinent positives in his symptomology as well as physiological changes evident through laboratory findings. Hence, it is necessary to understand and interpret each option in light of the patient's history, symptoms, and lab work. (A) Acute tubular necrosis (ATN) is a potential factor, given that the boy’s history of poor fluid intake can lead to ischemia and nephrotoxicity, triggering this condition. It's a form of acute kidney injury (AKI) typically characterized by elevated BUN and serum creatinine as well as an often abnormally high urinary sodium concentration. (B) Decreased intravascular volume could potentially result in prerenal AKI indicated by a relatively low urine sodium and high urine osmolality, which contrasts with his urinary laboratory results and, despite his history, would not explain the course. (C) Medication-related interstitial nephritis, mainly caused by drug-related hypersensitivity reactions, typical symptoms are rash, fever, and eosinophilia, which do not match his current symptoms. (D) Neurogenic bladder, a condition that occurs due to the brain injury causing impaired nerve signaling, could lead to urinary retention and possibly kidney damage over time. However, this does not explain his recent feverish symptoms. (E) Pyelonephritis might be considered due to rising temperatures and systemic signs of infection, but his urinalysis does not exhibit telltale leukocyte esterase or nitrites seen in urinary tract infections, making this unlikely. CLINICAL REASONING: Connecting the details and findings in this scenario, the patient has decreasing urine output along with high Blood Urea Nitrogen (BUN) and creatinine levels. High urinary specific gravity, hyponatremia, and hyperkalemia accompanying the mentioned symptoms indicate altered kidney function leading to oliguric renal failure. This kidney impairment can be due to prerenal, renal, or postrenal causes. His increased BUN and creatinine could suggest either a prerenal or intrinsic renal cause, but his fractional excretion of sodium (FeNa) (calculated as [urine Na × plasma creatinine] / [plasma Na × urine creatinine] × 100) being less than 1%, supports a prerenal cause more, pointing toward a decrease in effective circulatory volume triggering acute kidney injury. This could be from decreased intravascular volume due to poor fluid intake and additional losses through fever, rhinorrhea, and cough given his underlying condition, making option (B) more likely than the others. CONCLUDING: On a step-by-step analytical comparison of the choices in relation to the symptoms and findings, Decreased intravascular volume (B), by causing prerenal acute kidney injury, appears to have logical clinical support over other causes like Acute tubular necrosis, Medication-related interstitial nephritis, Neurogenic bladder, and Pyelonephritis, given the boy's specific condition and evidence provided. RESPONSE: Therefore, we can say that the most likely cause of this patient's altered kidney function is Decreased intravascular volume (Option B). This is based on his clinical picture indicating prerenal acute kidney injury as supported by his relative hypovolemia due to poor fluid intake and additional losses due to fever, rhinorrhea, and cough.

**Facilitator** **[Round 2]:** Summary: AI_A and AI_B both concluded that the most likely cause of the patient's altered kidney function is decreased intravascular volume. They both reasoned that the patient's symptoms and lab results, such as decreased urine output, increased BUN and creatinine, high urine specific gravity, and low urine sodium, point towards prerenal azotemia, a condition often seen due to decreased blood flow to the kidneys. AI_C, however, concluded that the most likely cause is pyelonephritis. They reasoned that the presence of respiratory symptoms, fever, and elevated WBC count, coupled with changes in kidney function, suggest a systemic infectious process that might have involved the kidneys. AI_D concluded that the most likely cause is a neurogenic bladder due to the patient's underlying neurological conditions. They reasoned that the patient's traumatic brain injury, developmental delay, and nonambulatory status could make him prone to development of a neurogenic bladder, where he cannot control his bladder function due to his neurological condition. AI_E agreed with AI_A and AI_B that the most likely cause is decreased intravascular volume. They reasoned that the patient's ongoing symptoms suggest a systemic illness causing decreased fluid intake or increased losses, leading to dehydration and thus decreased intravascular volume. Question to clarify disagreements: Given the patient's symptoms and lab results, what specific evidence leads you to conclude that the most likely cause of the patient's altered kidney function is decreased intravascular volume, pyelonephritis, or a neurogenic bladder? How do you rule out the other potential causes?. Based on your preceding response, how would you answer the original question: [A 10-year-old boy with a traumatic brain injury sustained during infancy is examined in the nursing care facility where he resides because he has had no urine output during the past 24 hours. During the past 4 days, he has had decreasing urine output, intermittent fever, rhinorrhea, and cough. As a result of his brain injury, the patient has severe developmental delay and a seizure disorder. He is fed orally, but he is nonverbal and nonambulatory. Medications are levetiracetam, docusate, and multivitamins. The patient is 131 cm (4 ft 2 in; 10th percentile) tall and weighs 26 kg (57 lb; 10th percentile). BMI is 14 kg/m² (10th percentile). Vital signs are temperature 38.8°C (101.8°F), pulse 80/min, respirations 25/min, and blood pressure 110/80 mm Hg. Pulse oximetry on room air shows an oxygen saturation of 95%. The patient appears thin. He is awake and is not in apparent distress. He is responsive to touch. Auscultation of the lungs discloses scattered coarse crackles and rhonchi but good air entry and normal work of breathing. Results of laboratory studies are shown: Serum Blood Urea nitrogen 78 mg/dL Creatinine 3.2 mg/dL Na+ 131 mEq/L K+ 5.6 mEq/L Cl− 88 mEq/L HCO³ −16 mEq/L Urine Specific gravity 1.030 (N=1.003–1.029) Creatinine 90 mg/dL Na+ 8 mEq/L Hematocrit 37% Hemoglobin 12.5 g/dL WBC 12,000/mm3 Neutrophils, segmented 60% Lymphocytes 40% Platelet count 250,000/mm3 Which of the following is the most likely cause of this patient's altered kidney function? (A) Acute tubular necrosis (B) Decreased intravascular volume (C) Medication-related interstitial nephritis (D) Neurogenic bladder (E) Pyelonephritis]

**AI_D:** Restating the Question: The question asks about the most probable cause of the observed change in the renal function of a 10-year-old boy presenting with no urine output over the past day and decreasing urine output over the past four days. The child has undergone a traumatic brain injury in infancy resulting in severe developmental delay coupled with seizure disorder. His basic metrics are at the 10th percentile with apparent thin physique and slight fever. His serum studies display elevations in BUN, creatinine, and potassium while his urine studies indicate higher specific gravity with decreased sodium. His WBC count is also somewhat high. Planning: Assuming the symptoms and lab reports, various conditions could be causing the patient's issue – acute tubular necrosis, a decrease in intravascular volume, drug-induced interstitial nephritis, neurogenic bladder, or pyelonephritis. We need to critically evaluate all these conditions and identify which one makes the most clinical sense in this specific case. Clinical Reasoning: Initially, considering the patient's trauma history and developmental issues, a neurogenic bladder might seem fitting. However, it is unlikely that bladder dysfunction would lead to the systemic alterations observed in this case, such as elevated BUN, creatinine, and low urine sodium. Acute tubular necrosis and drug-induced interstitial nephritis typically present with high urine sodium, and the patient's urinalysis does not match this. Pyelonephritis, a kidney infection, often has urinary symptoms and may cause changes in laboratory values, but typically it is associated with significantly higher fever and flank pain, which this child does not seem to have. Decreased intravascular volume, however, aligns with the boy's observed signs and lab results. The prolonged decreased urine output (oliguria) and high urine specific gravity indicate decreased water availability, increased water retention by kidneys, or pre-renal azotemia. Combined with raised serum BUN and creatinine, this suggests a likely decrease in renal perfusion secondary to reduced intravascular volume, and thus dehydration. Concluding: Given the patient's clinical presentation and lab findings, decreased intravascular volume appears to be the most likely cause of the patient's altered kidney function. Response: My response is (B) Decreased intravascular volume, is the most likely cause of this patient's altered kidney function.

**AI_B:** RESTATING THE QUESTION: The primary question is to identify the chief cause prompting this 10-year-old boy's altered kidney function. As per his medical history, he sustained traumatic brain injury during infancy, which resulted in several complications, such as severe developmental delay and a seizure disorder. He recently exhibited symptoms of reduced urine output, intermittent fever, rhinorrhea, and cough. The patient's laboratory results show elevated serum blood urea nitrogen (BUN), creatinine, and urine specific gravity, alongside increased neutrophils and white blood cell (WBC) count. PLANNING: The foremost assumption could be that the boy's neurological conditions, such as seizure disorder and nonambulatory status, could be leading to a neurogenic bladder resulting in altered kidney function. However, these can be questioned as they do not explain all the manifestations, particularly the systemic symptoms like fever and raised WBC count. An alternate assumption could be a systemic infection leading to pyelonephritis. However, although systemic symptoms and rise in WBC count support this, the low urine sodium and high urine specific gravity favor prerenal causes. Finally, the presence of symptoms suggestive of systemic illness causing decreased fluid intake or increased losses leading to dehydration and thus decreased intravascular volume should be clinically prioritized. CLINICAL REASONING: Considering these alternate assumptions, one could argue that decreased intravascular volume is a more viable option. The respiratory symptoms (manifested as rhinorrhea and cough), along with reduced fluid intake due to illness, could have led to dehydration, causing decreased intravascular volume. This might have caused prerenal azotemia, portrayed by the high BUN (>20:1) to creatinine ratio, high urine specific gravity (>1.020), and low urine sodium (<20 mEq/L). Additional evidence to this could be the absence of signs indicative of a more severe kidney injury, like muddy brown casts, which would suggest acute tubular necrosis (ATN), or new medications that could lead to interstitial nephritis. CONCLUDING: The boy's neurologic conditions, elevated BUN, creatinine, neutrophils, WBC count, high urine specific gravity, and low urine sodium seem to be interrelated. These manifestations occur due to a chain reaction initiated by systemic illness causing decreased intake or increased losses resulting in dehydration and decreased intravascular volume. RESPONSE: Thus, considering the clinical manifestations, I believe the most likely cause of the patient's altered kidney function is decreased intravascular volume (Choice B).

**AI_A:** RESTATING THE QUESTION: This question asks us to determine the cause of the altered kidney function in a 10-year-old boy who has experienced a decrease in urine output over the past few days and is showing signs of possible infection. The kidney function pertains to how well the kidneys are filtering waste from the bloodstream, which is reflected by such metrics as serum Blood Urea Nitrogen (BUN) and Creatinine. Additionally, other symptoms and the boy's medical history, including a traumatic brain injury, developmental delay, and a seizure disorder, are relevant in making this diagnosis. PLANNING: Before making a diagnosis, consider the following assumptions: 1) His altered kidney function is related to his recent acute symptoms (i.e., decreasing urine output, fever, rhinorrhea, and cough). 2) His kidney issues are directly linked to his underlying health issues due to his past traumatic brain injury including his seizure disorder and developmental delay. 3) His altered kidney function is due to his medications. By questioning these assumptions, we can generate alternate hypotheses. Moreover, clinical prioritization is needed here to achieve an accurate diagnosis. CLINICAL REASONING: The labs show an elevated BUN and creatinine, indicating a problem with the kidney's filtration function. Also, the high urine specific gravity and low urine sodium suggest that the kidneys are trying to conserve water, indicating volume depletion. This leads to the assumption that the boy's symptoms might be due to dehydration (decreased intravascular volume), perhaps caused by the boy's possible infection. His respiratory symptoms, fever, and elevated white blood cell count (which signify infection), might seem to point to pyelonephritis. However, this would not directly cause prerenal azotemia (indicative of volume depletion and decreased blood flow to the kidneys). Also, the urine studies don't indicate infection or inflammation, making this less likely. He does have a neurological disorder from his previous brain injury, which could cause a neurogenic bladder. This might lead to urinary retention and backflow to the kidneys, eventually affecting kidney function. However, the labs and vitals do not align with this scenario. Lastly, concerning the possibility of medication-induced interstitial nephritis, however, none of the patient's current medications are known to commonly cause this condition. CONCLUDING: Given the examination findings and lab results, among the choices provided, the most likely cause of this patient’s altered kidney function is decreased intravascular volume. The high urine-specific gravity and low urine sodium, along with the elevated BUN and creatinine, suggest that the kidneys are conserving water due to volume depletion. RESPONSE: Hence, regarding the original question concerning the cause of the patient's altered kidney function, my answer is (B) Decreased intravascular volume.

**AI_C:** REPHRASING THE QUESTION: This scenario centers around a 10-year-old boy who has significant medical issues stemming from a traumatic brain injury. He is currently facing a sudden decrease in urine output, along with symptoms such as fever, runny nose, and cough. Examination and laboratory results show an increased blood urea nitrogen (BUN) and creatinine, higher than normal urine specific gravity, and elevated white blood cell count. These findings indicate an altered kidney function and our task is to identify its most probable cause. The possible options are: Acute tubular necrosis, decreased intravascular volume, medication-related interstitial nephritis, neurogenic bladder, or pyelonephritis. PLANNING: The first assumption could be that the patient’s decreasing urine output and increased BUN and creatinine are due to acute renal failure. However, there could be other assumptions like a urinary tract infection leading to pyelonephritis, drug-induced kidney injury due to his medication, or decreased intravascular volume given his thin appearance and underlying conditions such as brain injury and nonambulatory status. Another assumption could be a neurogenic bladder - the boy's brain injury and subsequent developmental disability might have caused a dysfunction of his bladder leading to urinary retention. CLINICAL REASONING: His increased BUN and creatinine suggest a reduction in glomerular filtration rate (GFR), which can be caused by different pathological conditions, such as acute kidney injury, chronic kidney disease, or urinary tract obstruction. The fever, cough, and leukocytosis can be a nonspecific response to various disorders, or can specifically suggest an infectious process. The increased urea nitrogen and creatinine can occur in both urinary tract obstruction and renal parenchymal diseases, but the constellation of symptoms might be more suggestive of kidney disease. Acute tubular necrosis generally presents with oliguria or anuria, but the absence of risk factors such as ischemia or nephrotoxicity makes this a less probable assumption. Medication-related nephritis could occur with his medication, but it usually presents with rash, eosinophilia, or other systemic manifestations which are not mentioned in this case. Neurogenic bladder can cause urinary retention which can backfire on the kidneys and decrease their function, however, there are usually other signs such as bladder distension, recurring UTIs, or incontinence which are not reported in this case. CONCLUDING: With these considerations, the clinical manifestations of this patient seem to rather point toward volume depletion due to poor oral intake and potential increased losses due to fever and respiratory symptoms manifesting as pre-renal azotemia, rather than parenchymal kidney disease or obstruction. RESPONSE: Based on the clinical picture, underlying conditions, and the pattern of laboratory findings, the most probable cause of this patient's altered kidney function is (B) decreased intravascular volume.

**AI_E:** RESTATING THE QUESTION: This question aims to identify the root cause of the observed decline in kidney function in a 10-year-old male patient who sustained a traumatic brain injury during infancy. He has overwhelming pathognomonic symptoms such as decreased urine output, intermittent fever, and respiratory symptoms. He is nonambulatory and nonverbal due to his severe developmental delay, and is being medicated with levetiracetam, docusate, and multivitamins. His current health condition suggests a possible renal dysfunction, as identified in changes in his blood and urine parameters. The possible etiological factors include acute tubular necrosis, decreased intravascular volume, medication-related interstitial nephritis, neurogenic bladder, or pyelonephritis. PLANNING: In view of the varying perspectives of my fellow AI entities, I find that the key to solving this question lies in thorough analysis of the patient's signs, symptoms, and lab results. I assume the decreased urine output can be caused by acute kidney injury, which can be a result of either prerenal, intrarenal or postrenal etiologies. I will therefore consider all possible causes related to the renal dysfunction, while being mindful of the fact that the patient's baseline health status (severe developmental delay, history of brain injury, seizure disorder, thin body habitus) may affect the interpretation of his symptoms and lab findings. CLINICAL REASONING: Upon examination of his lab results, the patient has elevated BUN and creatinine levels, indicative of acute kidney injury (AKI). The disproportionately increased BUN relative to creatinine, along with the increased urine specific gravity and the low urine sodium (reflecting avid renal sodium reabsorption) suggest a prerenal etiology. Prerenal causes of AKI are due to a decrease in effective circulating volume leading to decreased renal perfusion. In this case, the patient's decreased oral intake (given his illness with fever, cough, and rhinorrhea leading to possible decreased feeding or increased losses from fever) and thin body habitus might have contributed to decreased intravascular volume. However, it is also worth considering other possible causes. While his medications could potentially cause interstitial nephritis, this condition usually presents with eosinophiluria, rash, and fever, which the patient lacks. Similarly, neurogenic bladder might be considered given his neurological history, however, it frequently manifests with urinary retention and overflow incontinence, neither of which is reported in this patient. Lastly, while pyelonephritis is a plausible cause, pyelonephritis usually presents with flank pain, fever, and urinary findings such as pyuria, which are not present in this case. CONCLUDING: Therefore, although this patient's presentation is complex and could lend itself to multiple interpretations, the most likely cause of his recent kidney function changes seems to be decreased intravascular volume given the evidence of prerenal azotemia on his lab findings and his clinical context. RESPONSE: The most likely cause of this patient's altered kidney function is (B) Decreased intravascular volume.

**Facilitator** The question asks for the most probable cause of altered kidney function in a 10-year-old boy with a history of traumatic brain injury, severe developmental delay, and a seizure disorder. The boy has been experiencing decreased urine output, intermittent fever, rhinorrhea, and cough. His lab results show elevated Blood Urea Nitrogen (BUN), creatinine, and urine specific gravity, along with increased neutrophils and white blood cell (WBC) count. The possible causes of the boy's altered kidney function include acute tubular necrosis, decreased intravascular volume, medication-related interstitial nephritis, neurogenic bladder, or pyelonephritis. However, the boy's symptoms and lab results do not align with all these conditions. For instance, acute tubular necrosis and medication-related interstitial nephritis typically present with high urine sodium, which the boy does not have. Pyelonephritis often presents with significantly higher fever and flank pain, which are also not reported in this case. Neurogenic bladder might be considered given his neurological history, but it frequently manifests with urinary retention and overflow incontinence, neither of which is reported in this case. On the other hand, the boy's symptoms and lab results align with decreased intravascular volume. The high urine specific gravity and low urine sodium, along with the elevated BUN and creatinine, suggest that the kidneys are conserving water due to volume depletion. This could be due to the boy's decreased oral intake and potential increased losses due to fever and respiratory symptoms. Therefore, based on the clinical picture, underlying conditions, and the pattern of laboratory findings, the consensus among the AI entities is that the most probable cause of this patient's altered kidney function is decreased intravascular volume.
